# Heterologous expression and characterization of xylose-tolerant GH 43 family β-xylosidase/α-L-arabinofuranosidase from *Limosilactobacillus fermentum* and its application in xylan degradation

**DOI:** 10.3389/fbioe.2025.1564764

**Published:** 2025-03-10

**Authors:** Robie Vasquez, Ji Hoon Song, Jae Seung Lee, Sanghoon Kim, Dae-Kyung Kang

**Affiliations:** Department of Animal Biotechnology, Dankook University, Cheonan, Republic of Korea

**Keywords:** beta-xylosidase, GH family 43, lactic acid bacteria, xylan degradation, xylose tolerant, xylo-oligosaccharide

## Abstract

The degradation of hemicellulose, including xylan, is an important industrial process as it provides cheap and sustainable source of economically valuable monosaccharides. β-xylosidases are key enzymes required for complete degradation of xylan and are used in the production of monosaccharides, such as xylose. In this study, we characterized a novel, xylose-tolerant β-xylosidase isolated from *Limosilactobacillus fermentum* SK152. Sequence analysis and protein structure prediction revealed that the putative β-xylosidase belongs to the glycoside hydrolase (GH) family 43 subfamily 11 and exhibits high homology with other characterised GH43 β-xylosidases from fungal and bacterial sources. The putative β-xylosidase was named *Lf*Xyl43. The catalytic residues of *Lf*Xyl43, which are highly conserved among GH 43 β-xylosidases, were predicted. To fully characterise *Lf*Xyl43, the gene encoding it was heterologously expressed in *Escherichia coli*. Biochemical characterisation revealed that the recombinant *Lf*Xyl43 (r*Lf*Xyl43) was active against artificial and natural substrates containing β-1,4-xylanopyranosyl residues, such as *p*-nitrophenyl-β-D-xylopyranoside (pNPX) and oNPX. Moreover, it demonstrated weak α-L-arabinofuranosidase activity. The optimal activity of r*Lf*Xyl43 was obtained at pH 7.0 at 35°C. r*Lf*Xyl43 could degrade xylo-oligosaccharides, such as xylobiose, xylotriose, and xylotetraose, and showed hydrolysing activity towards beechwood xylan. Moreover, r*Lf*Xyl43 demonstrated synergy with a commercial xylanase in degrading rye and wheat arabinoxylan. The activity of r*Lf*Xyl43 was not affected by the addition of metal ions, chemical reagents, or high concentrations of NaCl. Notably, r*Lf*Xyl43 exhibited tolerance to high xylose concentrations, with a *K*_i_ value of 100.1, comparable to that of other xylose-tolerant GH 43 β-xylosidases. To our knowledge, this is the first β-xylosidase identified from a lactic acid bacterium with high tolerance to salt and xylose. Overall, r*Lf*Xyl43 exhibits great potential as a novel β-xylosidase for use in the degradation of lignocellulosic material, especially xylan hemicellulose. Its high activity against xylo-oligosaccharides, mild catalytic conditions, and tolerance to high xylose concentrations makes it a suitable enzyme for industrial applications.

## 1 Introduction

Xylans are hemicelluloses with a β-D-xylanopyranosyl backbone, linked either via a β-1,4- or β-1,3-glycosidic bond (Li et al., 2023). Xylans with β-1,3-glycosidic bonds are found in some chlorophyte and red algae, while β-1,4-xylanopyranosyl backbone is the most predominant hemicelluloses in vascular plant cell walls (Moreira and Filho, 2016; Qaseem et al., 2025). Arabinose, glucuronic acid, ferulic acid, and *p*-coumaric acid are among the main side chains attached to the xylan backbone (Saha, 2003a; Moreira and Filho, 2016; Naidu et al., 2018; Li et al., 2023). Different plant sources contain different xylans, with varying types and degrees of side-chain substitutions (Saha, 2003a; Moreira and Filho, 2016; Naidu et al., 2018). Xylans are cheap and eco-friendly feedstock that are processed into valuable industrial and pharmacological products. Owing to the complexity of side-chain substitutions, complete degradation of xylans requires concerted activities of different enzymes including endo-β-1,4-xylanases, β-D-xylosidases, as well as debranching enzymes. Endo-xylanases hydrolyse the β-1,4 glycosidic bonds, freeing up xylo-oligosaccharides (XOS) of various lengths, whereas β-D-xylosidases attack these XOS to produce xylose as the end product (Rohman et al., 2019; Li et al., 2023). The activity β-D-xylosidases relieves the inhibitory effect of XOS on the endo-xylanase, resulting in more efficient breakdown of the xylan substrate. Xylose is an important product of xylan degradation, which can be converted to other valuable products, such as xylitol, ethanol, and lactate (Saha, 2003a). In addition, the degradation of xylan and XOS in the gastrointestinal tract via these enzymes has been reported to contribute to the intestinal health by promoting the production of beneficial short-chain fatty acids, such as butyrate, propionate, and acetate, by the commensal microbiome (Singh et al., 2015; Lin et al., 2016). This underscores the importance of xylan degradation, not only for industrial applications but also in promoting health.

β-Xylosidases (E.C. 3.2.1.37) are diverse enzymes varying in structures, hydrolytic activities, biochemical properties, and substrate preferences (Rohman et al., 2019; Li et al., 2023). According to the Carbohydrate-active Enzyme (CAZy) database, β-xylosidases are currently classified into 11 glycoside hydrolase (GH) families based on their structural properties: GH 1, 3, 5, 30, 39, 43, 51, 52, 54, 116, and 120. GH family 43 (GH 43) is the second largest GH family in the CAZy database, comprising not only of β-xylosidases but also other GH enzymes, such α-L-arabinofuranosidases, arabinases, and xylanases. The GH 43 family is further divided into 39 sub-families based on the structure of the C-terminal auxiliary domain (Rohman et al., 2019; Li et al., 2023). Unlike other β-xylosidases belonging to the other GH families, GH 43 β-xylosidases demonstrate an inverting mechanism (Rohman et al., 2019; Li et al., 2023). A number of GH 43 β-xylosidases and bi-functional β-xylosidases/α-L-arabinofuranosidases have been characterised from different bacterial and fungal sources. Aside from pH and thermal stability, robust nature, and high expression yield, which are valuable characteristics for industrial applications, some of these β-xylosidases have shown tolerance to high xylose concentrations. β-Xylosidases isolated from *Bacteroides ovatus* (Jordan et al., 2017), *Thermobifida halotolerans* YIM 90462^T^ (Yin et al., 2019), *Humicola insolens* Y1 (Yang et al., 2014), and *Enterobacter* sp. (Ontañon et al., 2018) are some of the reported GH 43 β-xylosidases with exceptionally high tolerance to xylose.

Lactic acid bacteria (LAB) form a diverse group of bacteria, generally used in food production, and are beneficial members of the intestinal microbiota in animal and human hosts (Axelsson and Ahrné, 2000; Bintsis, 2018). LAB include various species from *Lactobacillus, Pediococcus, Leuconostoc, Weisella, Oenococcus*, and *Streptococcus* among other genera (Axelsson and Ahrné, 2000; König and Fröhlich, 2017). Owing to their ecological niches, LAB are valuable sources of GH enzymes used for the degradation of plant biomass, including xylans and XOS (Vasquez et al., 2024). In fact, LAB can carry single or multiple copies of GH enzymes, including GH 43 β-xylosidase. Previous studies have demonstrated the capability of LAB to utilize XOS as carbon source indicating the presence and importance of β-xylosidases in their metabolic activities (Ohara et al., 2006; Moura et al., 2007; Jang and Kim, 2011; Maria et al., 2014; Iliev et al., 2020). However, β-xylosidases from LAB have been isolated and characterised in only a few studies. For instance, two β-xylosidases of the GH 43 family were identified, heterologously expressed, and characterised from *Levilactobacillus brevis* DSM 20054, and these exhibited activity towards XOS (Michlmayr et al., 2013). Falck et al. (2016) also reported a GH 43 β-xylosidase from *Weisella* sp. 92 that can hydrolyse short XOS. Similarly, a GH 43 β-xylosidase was identified from *Lactobacillus rossiae*, which could liberate xylose from XOS (Pontonio et al., 2016). Nevertheless, reports of xylose tolerant GH 43 β-xylosidases from LAB with xylan-degrading ability are scarce.

This study was aimed at investigating a putative GH 43 β-xylosidase, *Lf*Xyl43, identified from the genome of *Limosilatobacillus fermentum* SK152. We compared the molecular structure of *Lf*Xyl43 with those of other known GH 43 enzymes to elucidate its mechanism of action. Moreover, we heterologously expressed and characterised β-xylosidase, and determined its tolerance to xylose and salt. Finally, we explored the ability of this newly characterised β-xylosidase to degrade XOS and xylan.

## 2 Materials and methods

### 2.1 Bioinformatics analysis

Multiple sequence analysis of amino acid sequences of *Lf*Xyl43 and closely related GH 43 β-xylosidases/α-L-arabinofuranosidases was performed using UniProt Align (https://www.uniprot.org/align) after retrieving their sequences from the NCBI database (https://www.ncbi.nlm.nih.gov/). The phylogenetic tree was constructed using neighbour-joining method with Poisson correction in MEGA 11 (Tamura et al., 2021). Structural homology analysis and function prediction were performed using the Phyre 2.2 web server (Kelley et al., 2015). CAZy (https://www.cazy.org/) annotation and signal peptide identification was done via the dbCAN2 server (Zhang et al., 2018). The three-dimensional model of *Lf*Xyl43 was determined using AlphaFold2 (Jumper et al., 2021) via ColabFold (Mirdita et al., 2022). Protein–ligand docking was performed using CB-Dock2 (Liu et al., 2022), with *p*-nitrophenyl-β-D-xylopyranoside (pNPX; ChEBI ID 90148), xylobiose (ChEBI ID 28309), and xylotetraose (ChEBI ID 62972) used as ligands. Visualisation was performed using PyMol (ver. 3.0) (Schrödinger and DeLano, 2020).

### 2.2 Bacterial strains, plasmids, and culture conditions

All bacterial strains, plasmids, and primer sequences used in this study are summarised in Table 1. *Lm. fermentum* SK152 was cultured in Man Rogosa Sharpe (MRS) broth (BD Difco, United States) at 37°C, without aeration. For cloning and protein expression, *E. coli* DH5α and *Escherichia coli* BL21 (DE3) competent cells were used, respectively. *E. coli* strains were grown in Luria-Bertani (LB) broth (BD Difco, United States) supplemented with ampicillin (100 μg/mL) at 37°C, with aeration.

**TABLE 1:** Bacterial strains, plasmids, and primers used in this study.

	Features or sequences	Source
Strains		
*Escherichia coli* DH5α	Cloning host	Biofact
*E. coli* BL21 (DE3)	Expression host	Real BioTech
*Limosilactobacillus fermentum* SK152	Source of β-xylosidase gene, *Lfxyl43*	Yoo et al. (2017)
Plasmids		
pET21b (+)	Expression vector with 6× Histidine tag	Novagen
pET21-*Lf*Xyl43	pET21b (+) carrying 6× Histidine-tagged *Lfxyl43* gene	This study
Primers		
X1	GGG​GGG​AAG​CTTCAT​ATGAAA​ACT​ATC​CA AATCCG	This study
X2	AAA​TCT​AGACTC​GAGCCG​GCT​CGT​TAC​C	This study

Enzyme restriction sites are underlined accordingly: *Nde* I and *Xho* I.

### 2.3 Molecular cloning

The genomic DNA of *Lm. fermentum* SK152 was extracted following the protocol described by Yoo et al. (2017) and used as the template for amplifying the β-xylosidase/α-L-arabinofuranosidase gene (Supplementary Figure S1), *Lfxyl43*, using the primers listed in Table 1. PCR was performed following the method described by Vasquez et al. (2022). For constructing the expression vector, the amplified *Lfxyl43* gene was digested using the restriction enzymes *Nde*I and *Xho*I (TaKaRa, Tokyo, Japan), and then cloned into the pET21b (+) (Novagen) vector using T4 ligase (TaKaRa, Tokyo, Japan). The resulting expression vector with the *Lfxyl43* gene was transformed into *E. coli* DH5α to verify the sequence, and into *E. coli* BL21 (DE3) for protein overexpression.

### 2.4 Protein expression and purification

For expressing the 6× histidine-tagged recombinant *Lf*Xyl43 (r*Lf*Xyl43), transformed *E. coli* BL21 (DE3) was grown overnight in LB broth (with ampicillin, 100 μg/mL final concentration) at 37°C, with shaking. The culture was then diluted at 1:100 in LB broth with ampicillin and allowed to grow to an optical density at 600 nm (O.D._600_) of 0.5 before induction with 0.1 mM isopropyl- β-D-thiogalactopyranoside (IPTG). After IPTG addition, the culture was incubated at 20°C for 12 h, with aeration. The cells were harvested by centrifugation (10,000 × *g* for 10 min) and the pellet, thus obtained, was washed twice with 1× phosphate-buffered saline (PBS). The cells were resuspended in 50 mM sodium phosphate buffer (pH 7.0) and sonicated for 6–7 cycles (10 s sonication, 15 s pause) on ice. Cell-free extract was collected by centrifugation (15,000 × *g* for 20 min at 4°C) and then filtered through a 0.22 µm filter to remove cell debris. For purifying the recombinant protein, the cell-free extract was applied to an equilibrated nickel-nitrilotriacetic acid (Ni-NTA) resin (Qiagen, Hilden, Germany) for 1 h at 4°C. The protein was eluted with 50 mM sodium phosphate buffer containing 250 mM imidazole. The purified protein was dialysed against 50 mM sodium phosphate buffer with 20% glycerol (pH 7.0) using a dialysis membrane (30,000 MWCO). The concentration of purified protein was determined using the Bradford assay, with bovine serum albumin used as a standard. The purified proteins were stored at −20°C until further use. Sodium dodecyl sulphate-polyacrylamide gel electrophoresis (SDS-PAGE, 12%) was performed according to Vasquez et al. (2022) to verify expression and purification. The protein marker (MW: 10–245 kDa) used in this study was purchased from LPS Solution (Daejon, Republic of Korea).

### 2.5 Enzyme activity assay

For measuring the enzymatic activity of r*Lf*Xyl43, the method by Falck et al. (2016) was followed, with some modifications. Briefly, the standard enzyme assay was performed at 35°C for 5 min in 50 mM sodium phosphate buffer (pH 7.0) with 10 µg purified r*Lf*Xyl43 and 2 mM pNPX (Sigma, MO, United States). The reaction was stopped by adding an equal volume of 1 M Na_2_CO_3_. The release of *p*-nitrophenol was measured spectrophotometrically at 405 nm (SpectraMax, Molecular Diagnostics, United States). One unit of enzyme activity was defined as the amount of enzyme needed to release 1 μmol of *p*-nitrophenol per minute under the assay conditions. All assays were performed in triplicates.

### 2.6 Biochemical characterisation

The optimum pH for the β-xylosidase and α-L-arabinofuranosidase activity of r*Lf*Xyl43 was determined by performing the standard enzyme assay at different pH (2.0–10.0) (Supplementary Table S1). While the optimal temperature was determined by performing the standard enzyme assay at different temperatures (20°C–80°C). The substrates used were pNPX or *p*-nitrophenyl-α-L-arabinofuranoside (pNPAf; Sigma, MO, United States). The effect of pH on the activity of r*Lf*Xyl43 was assessed by pre-incubating the enzyme at different pH (2.0–10.0) in the absence of the substrate at 20°C for 1 h. The substrate was added after the pre-incubation, and then the standard assay was performed. The thermal stability of r*Lf*Xyl43 was measured by pre-incubating the enzyme (in 50 mM sodium phosphate buffer, pH 7.0) without the substrate at different temperatures (25, 30, 35, 40, and 45°C) for 0, 30, 60, 90, and 120 min. After the pre-incubation, the mixtures were cooled before adding the substrate. The standard enzyme assay was performed as described above.

The substrate specificity of r*Lf*Xyl43 was determined by performing the standard assay with different *p*-nitrophenyl or *o*-nitrophenyl substrates (2 mM), including pNPX, pNPAf, and *o*-nitrophenyl-β-D-xylopyranoside (oNPX; Sigma, MO, United States). All assays were performed in triplicates.

The activity of r*Lf*Xyl43 towards XOS, xylans, and arabinoxylan was measured by performing the standard assay using 2 mM xylobiose, xylotriose, or xylotetraose (Megazyme, Ireland) or 1% (w/v) beechwood xylan (Serva, Germany), rye or wheat arabinoxylan (Megazyme, Ireland) in 50 mM sodium phosphate buffer (pH 7.0). The reaction mixture was incubated at 35°C for 30 min. The release of reducing sugar was measured by adding an equal volume of 3, 5-dinitrosalycylic acid (DNSA), followed by boiling for 5 min (Miller, 1959). The reaction tubes were cooled down and the absorbance was measured at 540 nm. One unit of enzyme activity was defined as the amount of enzyme needed to release 1 µmol of reducing sugar equivalent to xylose per minute under the assay conditions. All assays were performed in triplicates.

The kinetic parameters (*K*_m_ *, V*_max_ *, k*_cat_, and *k*_cat_ */K*_m_) for r*Lf*Xyl43 were determined by performing the standard enzyme assay using different concentrations of pNPX, pNPAf, or XOS (1–10 mM). The kinetic parameters were calculated by fitting the non-linear regression to the Michaelis–Menten equation in GraphPad Prism for Windows (ver. 10.0). All assays were performed in triplicates.

The effect of metal ions and other chemical reagents on the activity of r*Lf*Xyl43 was determined by individually adding various metal ion solutions (K^+^, Ca^2+^, Fe^3+^, Mg^2+^, Mn^2+^, and Ni^2+^) or chemical reagents (EDTA, SDS, and DTT) to the reaction mixture at a final concentration of 1 or 10 mM. Standard assays were performed as previously described and the values were compared with those for the control without any addition.

The tolerance of r*Lf*Xyl43 to xylose and NaCl was evaluated according to Yang et al. (2014), with some modifications. Briefly, the enzyme was pre-incubated in increasing concentrations of xylose or NaCl (0–1,500 mM) at 35°C for 30 min before adding the substrate (2 mM pNPX). The standard enzyme assay was then performed to measure the residual activity. The competitive inhibitor constant, *K*_i_, for xylose was then calculated and defined as the amount of xylose required to inhibit half of the β-xylosidase activity under the assay conditions.

### 2.7 Hydrolysis of xylo-oligosaccharides, xylan, and arabinoxylans

The degree of synergy between r*Lf*Xyl43 and a commercial xylanase from *Trichoderma viridae* (Sigma, MO, United States) was determined by reacting 1 U/mL of r*Lf*Xyl43 alone or xylanase alone, or in combination with 1% (w/v) beechwood xylan, rye, or wheat arabinoxylan in 50 mM sodium phosphate buffer (pH 7.0). Each reaction mixture was incubated at 35°C for 30 min. The activity was measured as described above using the DNSA method. The degree of synergy was defined as the ratio of released xylose when the assay was performed with the two enzymes simultaneously to the sum of released xylose when the assay was performed with each enzyme alone. Thin layer chromatography (TLC) was performed by double ascending the samples on a silica gel F_254_ plates (Merck, MA, United States) using 1-butanol:acetic acid:water (2:1:1, v/v/v) as the mobile phase. The migration of hydrolysis products was detected by spraying diphenylamine in aniline:acetone:phosphoric acid solution (2:2:100:15, w/v/v/v) onto the plates, followed by heating until the bands appeared.

### 2.8 Statistical analysis

All data in this study were reported as mean ± standard deviation (SD) of triplicate experiments. The SD was less than 5%.

## 3 Results

### 3.1 Sequence analysis

**FIGURE 1:**
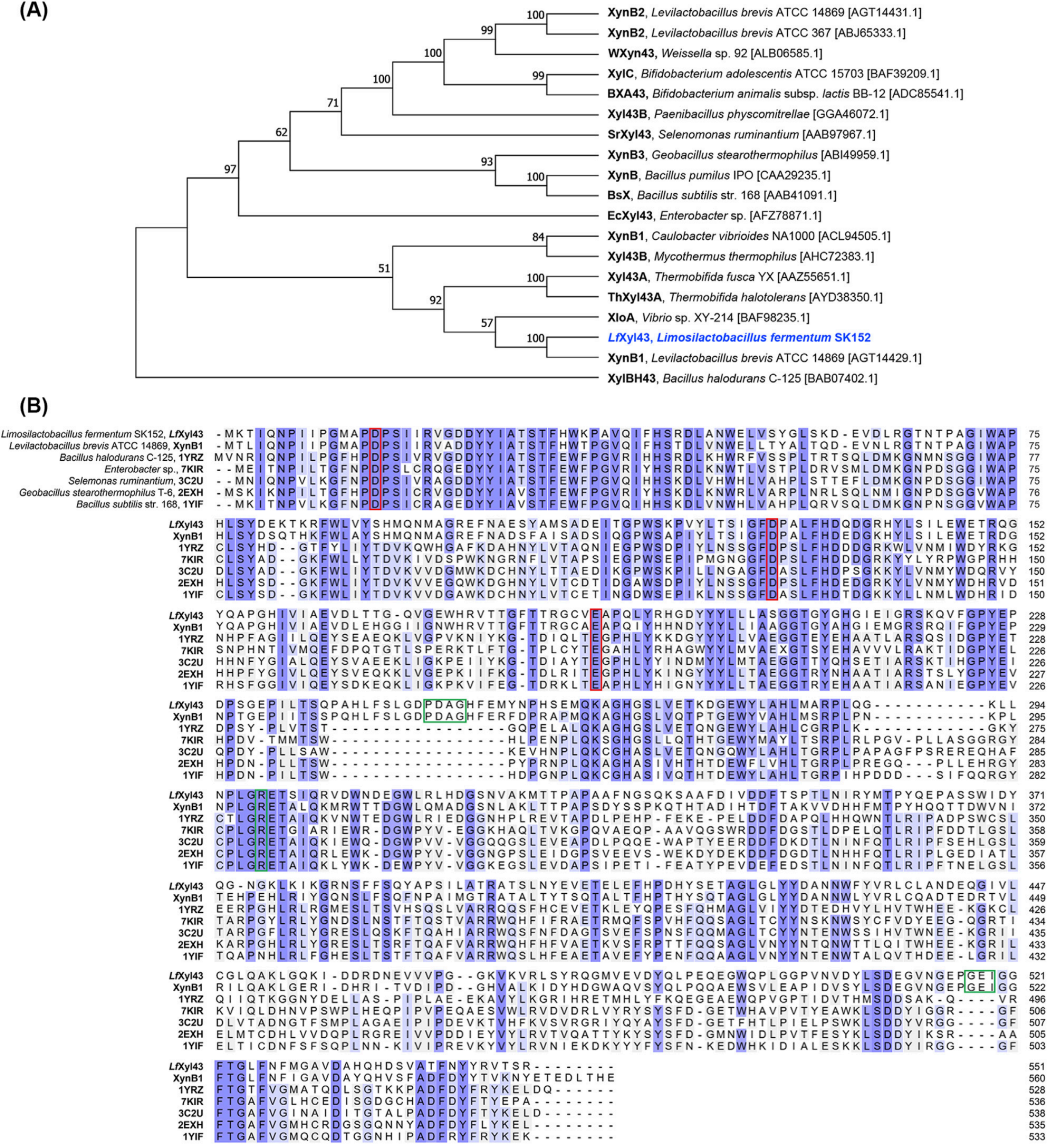
Sequence analysis of the β-xylosidase/α-L-arabinofuranosidase, *Lf*Xyl43, from *Limosilactobacillus fermentum* SK152. Phylogenetic tree showing the relationship of the amino acid sequence of *Lf*Xyl43 with those of other glycoside hydrolase (GH) 43 subfamily 11 enzymes **(A)**. Reference sequences were retrieved from NCBI GenBank. The phylogenetic tree was built using neighbour-joining method with bootstrap analysis of 1,000 and Poisson correction method in MEGA 11. Bootstrap values (in %) are indicated at each node. Multiple sequence alignment of the amino acid sequence of *Lf*Xyl43 against those of other GH 43 β-xylosidases and α-L-arabinofuranosidases **(B)**. Conserved sequences are shaded in blue. The putative catalytic residues are indicated with the red box, while the predicted contact residues are indicated in green box.

The *Lfxyl43* gene, which contains 1,656 base pairs and encodes a 551-amino acid protein (Supplementary Figure S2), was identified from the genome of *Lm. fermentum* SK152 (GenBank Accession No. PQ818275). The gene does not contain any signal peptides, suggesting that the encoded protein is cytoplasmic. Annotation of the amino acid sequence using the CAZy database via dbCAN2 predicted that the encoded protein, *Lf*Xyl43, belongs to the GH 43 subfamily 11. Phylogenetic analysis (Figure 1A) with other GH 43 subfamily 11 members revealed that *Lf*Xyl43 had high sequence similarity (67.6%) with XynB1 from *L. brevis* ATCC 14869 compared to other enzymes belonging to the same subfamily. Preliminary analysis of the tertiary structure and function of the protein using the Phyre 2.2 server revealed high structural homology of *Lf*Xyl43 with GH 43 enzymes, including β-xylosidase from *Bacillus halodurans* C-125 (PDB ID: 1YRZ), *B. subtilis* (PDB ID: 1YIF), and *Geobacillus stearothermophilus* T-6 (PDB ID: 2EXH), among others. The GH 43 β-xylosidase from *B. halodurans* C-125 (PDB ID: 1YRZ) is reported to have three highly conserved catalytic residues: Asp16, Asp129, Glu188. MSA analysis showed that *Lf*Xyl43, together with other characterised β-xylosidases, contains these highly conserved residues (Figure 1B). The amino acid residues Pro249, Asp250, Ala251, Gly252, Arg299, Gly517, Glu518, Ile519, and Phe522 were predicted to have key roles in substrate binding.

**FIGURE 2:**
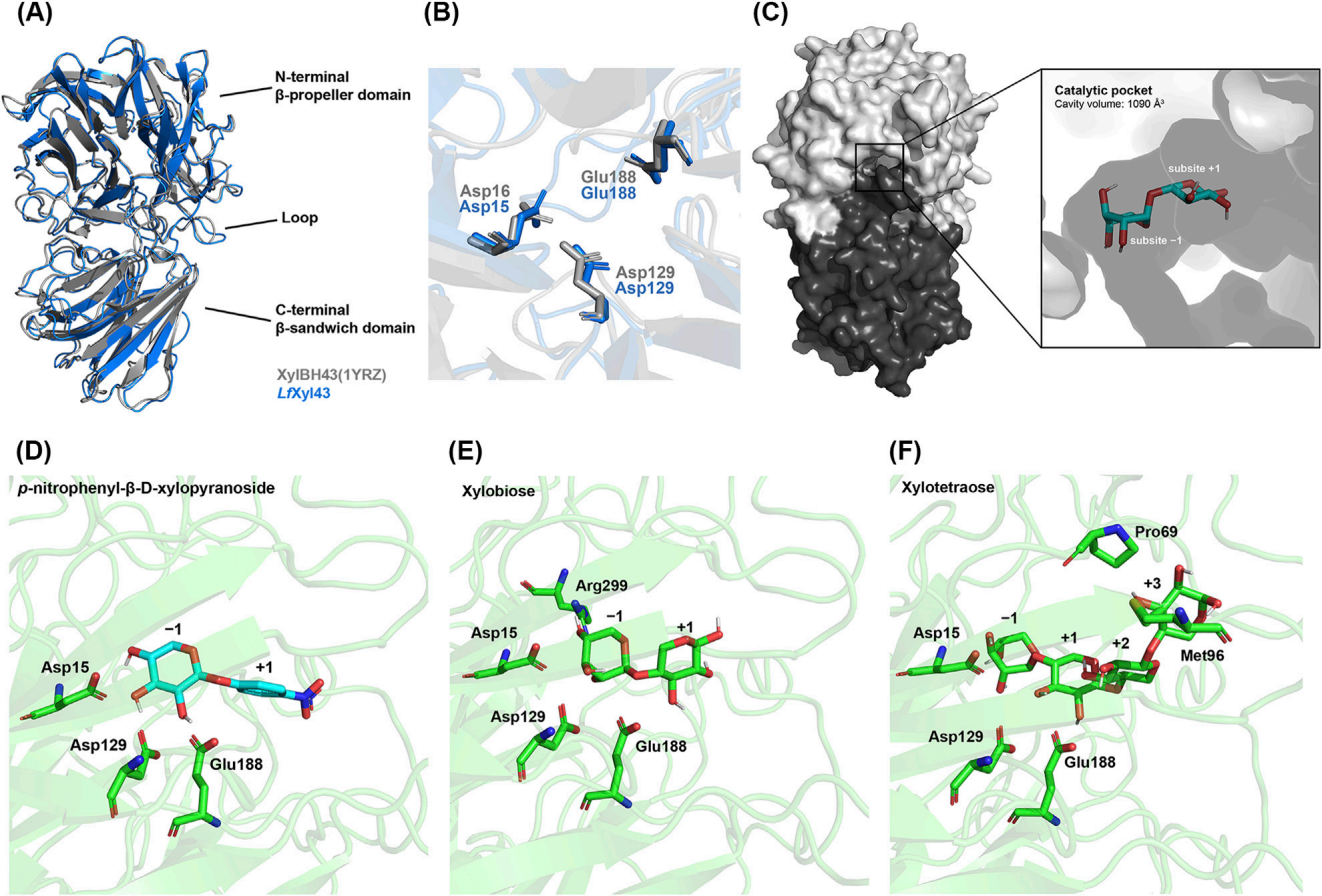
Three-dimensional model of β-xylosidase/α-L-arabinofuranosidase, *Lf*Xyl43, from *Limosilactobacillus fermentum* SK152 and its interactions with its ligands. Alignment of the predicted tertiary structure of *Lf*Xyl43 (blue) with that of XylBH43 (gray) from *Bacillus halodurans* C-125 (PDB ID: 1YRZ) showing the N-terminal 5-bladed β-propeller and the auxiliary β-sandwich domain connected via the loop **(A)**. Alignment of the catalytic residues of *Lf*Xyl43 (blue) and XylBH43 (gray) **(B)**. Surface representation of *Lf*Xyl43 and the location of the catalytic pocket complexed with a ligand at sub-sites −1 and +1 **(C)**. The putative catalytic residues Asp15, Asp129, and Asp188 and their predicted poses relative to the ligands: *p*-nitrophenyl-β-D-xylopyranoside **(D)**, xylobiose **(E)**, and xylotetraose **(F)**. See the main text for the Vina score for each protein–ligand complex.

For further studying the molecular structure of the putative β-xylosidase *Lf*Xyl43, its three-dimensional structure was modelled using AlphFold2 via ColabFold. Alignment of the structure of *Lf*Xyl43 with that of XylBH43 of *B. halodurans* C-125 (PDB ID: 1YRZ) revealed 94% homology (Figure 2A). In addition, the tertiary structure of *Lf*Xyl43 showed an N-terminal five-bladed β-propeller domain (Ile4 to Trp313), a C-terminal β-sandwich domain (Asp346 to Val548), and a loop (Leu314 to Val345) connecting the two structural domains (Figure 2A). Moreover, the catalytic residues of 1YRZ aligned with Asp15, Asp129, and Glu188 (Figure 2B), suggesting that these residues are responsible for the catalytic activity of *Lf*Xyl43. The putative catalytic pocket was predicted using CB-Dock2 to be positioned within the N-terminal β-propeller domain, with a predicted volume of 1,090 Å^3^ (Figure 2C). The catalytic pocket was complexed with xylobiose to show subsites −1 and +1. Protein–ligand docking was also performed to predict the interaction of *Lf*Xyl43 with its potential ligands. Figures 2D–F show the predicted position of pNPX (Vina score: −8.2), xylobiose (Vina score: −6.9), and xylotetraose (Vina score: −7.9) relative to the putative catalytic residues and other contact residues.

### 3.2 Heterologous expression and purification of r*Lf*Xyl43

**FIGURE 3:**
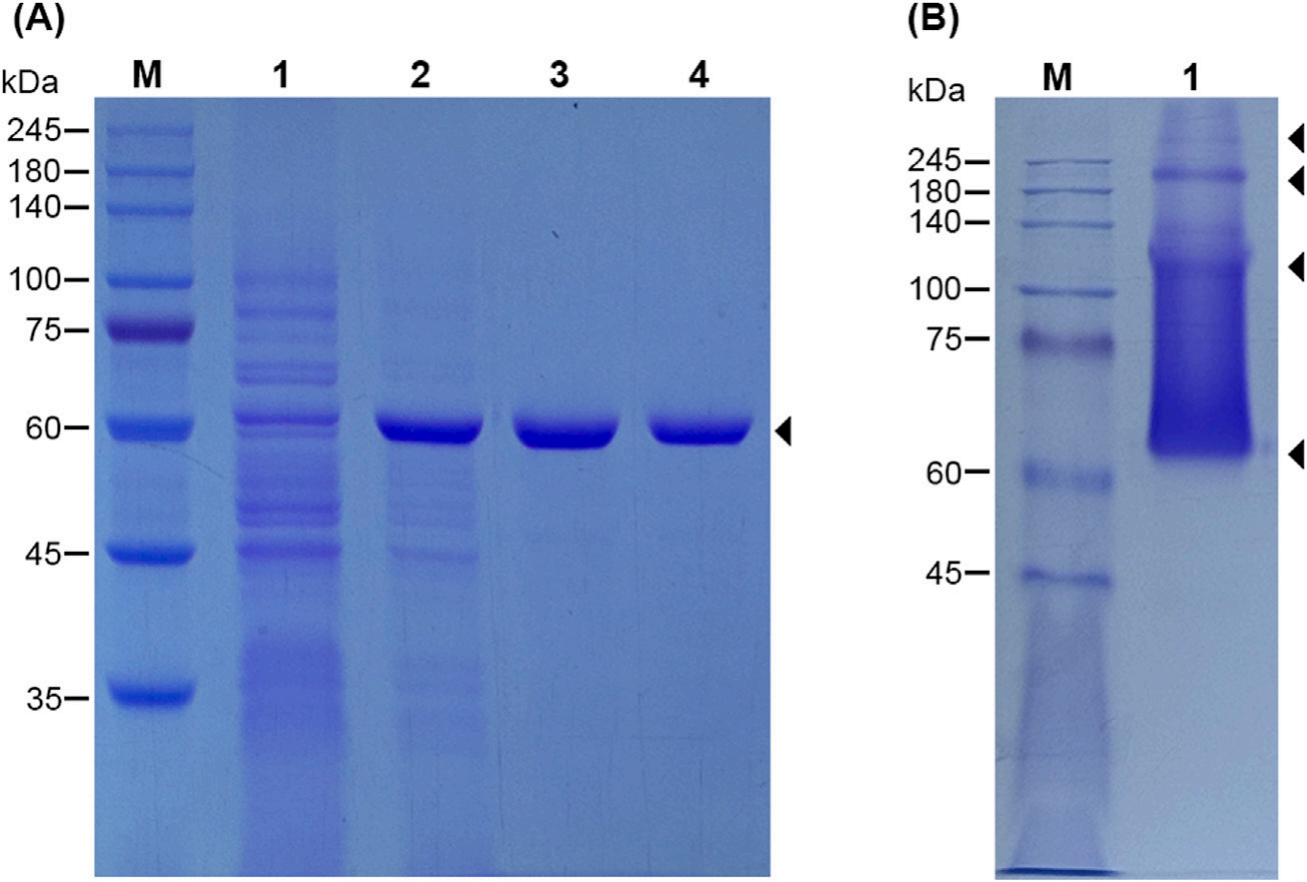
SDS-PAGE analysis of the recombinant β-xylosidase/α-L-arabinofuranosidase, r*Lf*Xyl43, from *Limosilactobacillus fermentum* SK152 **(A)**. Lane M, protein marker (range 10–245 kDa); lane 1, cell-free extract of uninduced *Escherichia coli* BL21 (DE3); lane 2, cell-free extract of IPTG-induced *E. coli* BL21 (DE3) after overexpression for 12 h at 20°C; lane 3, Ni-NTA-purified r*Lf*Xyl43; lane 4, r*Lf*Xyl43 after dialysis. The protein size is 62.5 kDa (including the 6× histidine tag). Native-PAGE analysis r*Lf*Xyl43 showing possible oligomeric states of the protein **(B)**. Lane M, protein marker; lane 1, purified r*Lf*Xyl43 in native PAGE loading buffer.

For further studying the activity of *Lf*Xyl43, the *Lfxyl43* gene was successfully cloned into the pET21b expression vector using the restriction enzymes *Nde*I and *Xho*I to produce a recombinant protein with a 6× histidine tag at its C-terminus. After overexpression, the enzyme was expressed as soluble protein and found mostly in the cell-free extract of *E. coli* BL21 (DE3) (Figure 3). The crude protein from the cell-free extract of *E. coli* was then purified using Ni-NTA agarose resin and dialysed against a protein storage buffer. r*Lf*Xyl43 was highly expressed, with a total protein yield of 121.2 mg/L of the original culture (1,138 U). To confirm its size, the purified protein was loaded on a 12% SDS-PAGE gel. Based on the amino acid sequence, the protein with the 6× histidine tag was found to have an expected size of 62.5 kDa, which was consistent with the SDS-PAGE result showing a single band at around the expected size (Figure 3A). To determine the oligomeric state of r*Lf*Xyl43, the purified protein was loaded on a native PAGE gel (Figure 3B), which after electrophoresis showed multiple bands with sizes exceeding 62 kDa, indicating that r*Lf*Xyl43 may exist in dimeric, trimeric, or tetrameric form.

### 3.3 Biochemical characterisation of r*Lf*Xyl43

**FIGURE 4:**
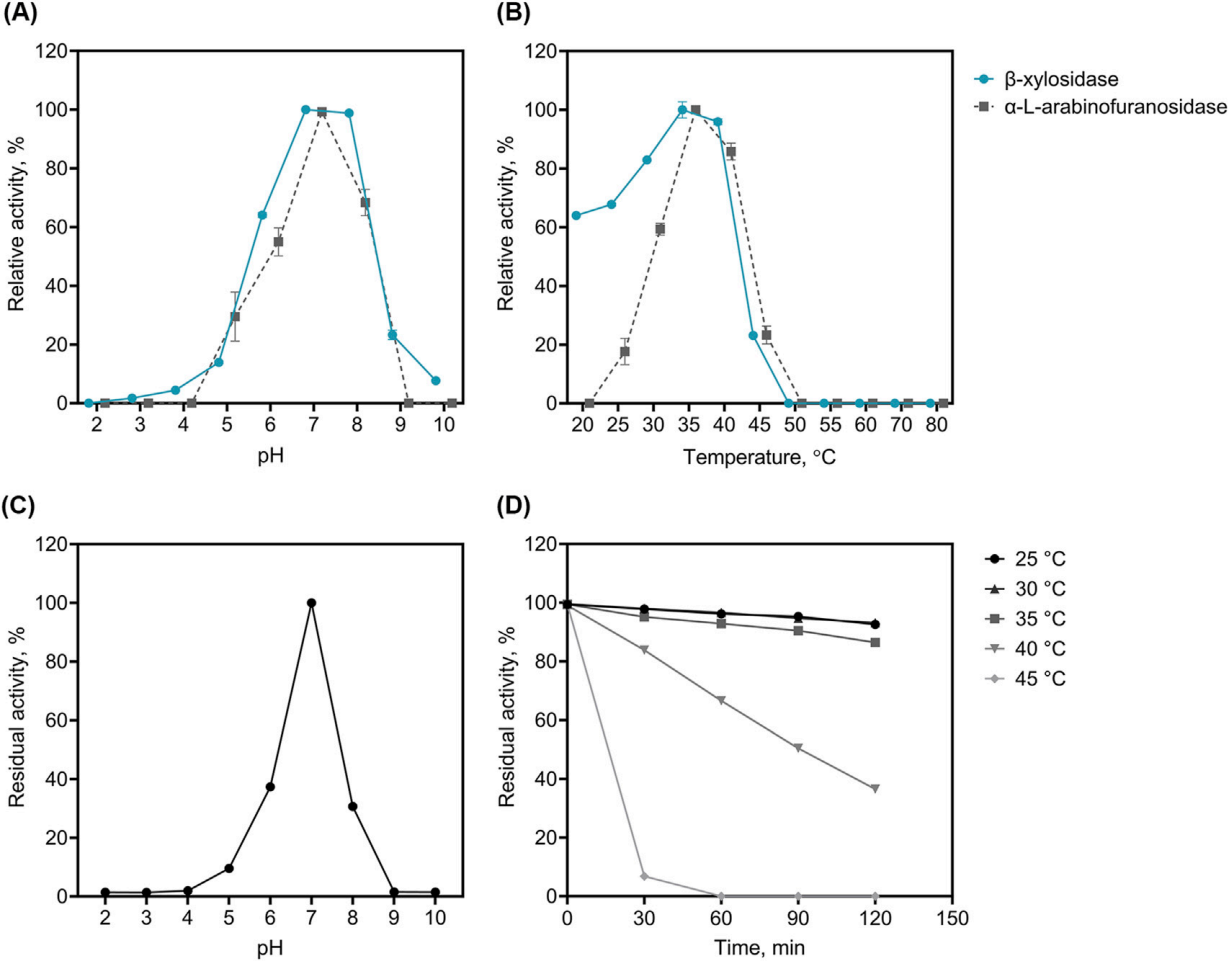
Biochemical characterization of the recombinant β-xylosidase/α-L-arabinofuranosidase, r*Lf*Xyl43, from *Limosilactobacillus fermentum* SK152. The optimal pH **(A)** and temperature **(B)** of r*Lf*Xyl43 determined using either *p*-nitrophenyl-β-D-xylopyranoside or *p*-nitrophenyl-α-L-arabinofuranoside. The stability of r*Lf*Xyl43 at different pH **(C)** was measured after 1 h incubation of the enzyme without the substrate in buffers with different pH. The thermal stability **(D)** of r*Lf*Xyl43 was determined by incubating the enzyme at different temperatures (25, 30, 35, 40, and 45°C) and the residual activity was measured at specific time points. Data are reported as the mean ± standard deviation of triplicate experiments. Some error bars smaller than the symbol are not shown.

We performed enzyme assays for biochemical characterisation of r*Lf*Xyl43 (Figure 4). r*Lf*Xyl43 showed β-xylosidase and α-L-arabinofuranosidase activities over a pH range from 5.0 to 9.0, with the highest β-xylosidase activity observed between pH 7.0 and 8.0, and the highest α-L-arabinofuranosidase activity noted at pH 7.0 (Figure 4A). At the optimal pH (7.0), r*Lf*Xyl43 showed β-xylosidase activity from 20°C to 45°C, with maximum activity at 35°C (Figure 4B). Moreover, the α-L-arabinofuranosidase activity of r*Lf*Xyl43 was observed from 25°C to 45°C, with an optimum at 35°C (Figure 4B). The recombinant *Lf*Xyl43 showed no β-xylosidase or α-L-arabinofuranosidase activity at temperatures higher than 50°C. It showed residual activity after pre-incubation at pH 5.0 to 8.0 but was most stable at pH 7.0 (Figure 4C). The thermal stability of r*Lf*Xyl43 was also determined (Figure 4D). The enzyme retained approximately 90% of its activity after 120 min at 25, 30, and 35°C but gradually lost its activity to 40% after 120 min at 40°C. Finally, r*Lf*Xyl43 showed loss in residual activity after 30 min at 45°C.

### 3.4 Substrate specificities and kinetic parameters of r*Lf*Xyl43

The substrate specificities of r*Lf*Xyl43 were determined using different artificial substrates, XOS, xylans, and arabinoxylans (Table 2). r*Lf*Xyl43 showed high activity towards pNPX and oNPX, with specific activities of 9.4 and 3.7 U/mg, respectively. It also showed weak activity (0.7 U/mg) towards pNPAf, indicating that β-xylosidase is the dominant activity of r*Lf*Xyl43. By contrast, r*Lf*Xyl43 showed no activity against other artificial substrates. r*Lf*Xyl43 showed high hydrolytic activities towards xylobiose (35.8 U/mg), xylotriose (25.3 U/mg) and xylotetraose (30.2 U/mg). Conversely, the enzyme demonstrated weak activity towards xylans (0.7 U/mg for beechwood xylan) and no detectable activity towards rye or wheat arabinoxylans.

**TABLE 2:** Substrate specificities of recombinant β-xylosidase/α-L-arabinofuranosidase, r*Lf*Xyl43, from *Limosilactobacillus fermentum* SK152.

Substrates	Specific activity, U/mg
*p*-nitrophenyl-β-D-xylopyranoside (pNPX)	9.4 ± 0.1
*p*-nitrophenyl-α-D-xylopyranoside	n.d.
*p*-nitrophenyl-α-L-arabinofuranoside (pNPAf)	0.7 ± 0.1
*p*-nitrophenyl-α-L-arabinopyranoside	n.d.
*p*-nitrophenyl-β-D-fucopyranoside	n.d.
*p*-nitrophenyl-β-D-galactopyranoside	n.d.
*p*-nitrophenyl-α-D-galactopyranoside	n.d.
*p*-nitrophenyl-α-D-glucopyranoside	n.d.
*p*-nitrophenyl-β-D-glucopyranoside	n.d.
*p*-nitrophenyl-α-D-mannopyranoside	n.d.
*p*-nitrophenyl-β-D-mannopyranoside	n.d.
*p*-nitrophenyl-α-L-rhamnopyranoside	n.d.
*o*-nitrophenyl-β-D-xylopyranoside (oNPX)	3.8 ± 0.1
*o*-nitrophenyl-β-D-galactopyranoside	n.d.
*o*-nitrophenyl-β-D-glucopyranoside	n.d.
Xylobiose	35.8 ± 3.4
Xylotriose	25.3 ± 1.5
Xylotetraose	30.2 ± 1.3
Beechwood xylan	0.7 ± 0.2
Rye arabinoxylan	n.d.
Wheat arabinoxylan	n.d.

Values are reported as mean ± standard deviation of triplicate experiments.

n.d., not detected.

The kinetic constants for the hydrolysis of pNPX, pNPAf, and XOS were determined and are summarized in Table 3. The apparent *K*_m_ for the artificial substrates pNPX and pNPAf was 2.0 and 1.5 mM and *V*_max_ was 19.7 and 0.4 μmol min^−1^ mg^−1^, respectively. The turnover rate (*k*_*c*at_) for pNPX and pNPAf was 141.8 and 2.8 s^−1^ and the catalytic efficiency (*k*_cat_/*K*_m_) was 7.2 and 1.9 s^−1^ mM^−1^, respectively, demonstrating higher catalytic efficiency with pNPX than with pNPAf. r*Lf*Xyl43 demonstrated increasing *K*_m_ values towards longer XOS (xylobiose < xylotriose < xylotetraose), indicating higher affinity for XOS with higher degree of polymerization. Similarly, the enzyme also demonstrated higher turnover and catalytic efficiency towards xylotetraose (3,526 s^−1^ and 41.9 s^−1^ mM^−1^) than towards xylobiose (3.2-fold) or xylotriose (1.6-fold).

**TABLE 3:** Kinetic parameters of recombinant β-xylosidase/α-L-arabinofuranosidase, r*Lf*Xyl43, from *Limosilactobacillus fermentum* SK152.

Substrates	*K*_m_ (mM)	*V*_max_ (µmol min^−1^ mg^−1^)	*k*_cat_ (s^−1^)	*k*_cat_/*K*_m_ (s^−1^ mM^−1)^
pNPX	2.0	19.7	141.8	7.2
pNPAf	1.5	0.4	2.8	1.9
Xylobiose	134.7	241.2	1741	12.9
Xylotriose	120.2	440.7	3,173	26.4
Xylotetraose	84.2	487.7	3,526	41.9

### 3.5 Effect of metal ions and other reagents on the activity of r*Lf*Xyl43

The effect of ions and other reagents on the activity of r*Lf*Xyl43 was also investigated (Table 4). The addition of 1 mM ions to the reaction mixture had little effect on the β-xylosidase activity of r*Lf*Xyl43. The addition of Ca^2+^, Mn^2+^, Mg^2+^, and K^+^ increased the residual activity of r*Lf*Xyl43 slightly. At 10 mM, the presence of Ca^2+^, Fe^3+^, Mn^2+^, Mg^2+^, and K^+^ reduced the activity of r*Lf*Xyl43, whereas Ni^2+^ dramatically weakened the hydrolysis of pNPX to 68.6%. The presence of 1 or 10 mM EDTA and SDS did not drastically affect the activity of r*Lf*Xyl43 towards pNPX. However, DTT reduced its activity to 85.4% at 1 mM and to 72.9% at 10 mM.

**TABLE 4:** Effect of metal ions and other reagents on the activity of recombinant β-xylosidase/α-L-arabinofuranosidase, r*Lf*Xyl43, from *Limosilactobacillus fermentum* SK152.

Ions or reagents	Residual activity, %	
	1 mM	10 mM
Control	100.0	100.0
Ca^2+^	102.1 ± 1.1	94.6 ± 1.0
Fe^3+^	99.6 ± 1.0	96.3 ± 0.2
Mn^2+^	103.0 ± 1.3	98.5 ± 0.9
Mg^2+^	100.9 ± 1.3	98.3 ± 0.8
Ni^2+^	96.0 ± 1.7	68.6 ± 0.2
K^+^	102.1 ± 1.2	96.3 ± 0.6
Ethylenediaminetetraacetic acid (EDTA)	99.7 ± 1.2	99.9 ± 0.8
Sodium dodecyl sulphate (SDS)	100.2 ± 2.2	99.9 ± 0.8
Dithiothreitol (DTT)	85.4 ± 0.8	72.9 ± 0.8

Values are reported as mean ± standard deviation of triplicate experiments.

### 3.6 Determination of the tolerance of r*Lf*Xyl43 to xylose and NaCl

**FIGURE 5:**
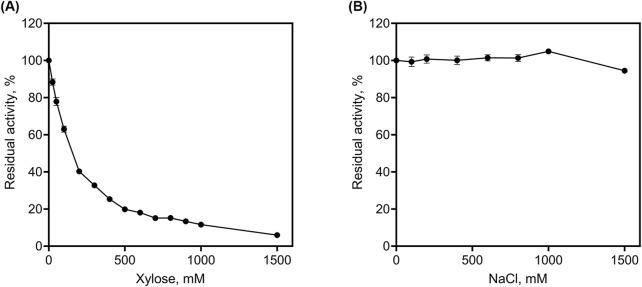
Determination of tolerance of the recombinant β-xylosidase/α-L-arabinofuranosidase, r*Lf*Xyl43, from *Limosilactobacillus fermentum* SK152 to xylose **(A)** and NaCl **(B)**.

We determined the tolerance of r*Lf*Xyl43 to xylose (Figure 5A). At 100 mM xylose, r*Lf*Xyl43 retained 60% of its activity towards pNPX. At 500 mM xylose, r*Lf*Xyl43 still exhibited 20% activity relative to the control and eventually decreased to 6% at 1,500 mM xylose. The calculated *K*_i_ for xylose as a competitive inhibitor was 100.1 mM. Table 5 shows the reported *K*_i_ values for other GH 43 enzymes from bacterial or fungal sources. r*Lf*Xyl43 showed higher tolerance to xylose compared to the β-xylosidases/α-L-arabinofuranosidases from *Lv. brevis* ATCC 367, *Fusarium proliferatum*, *B. ovatus*, *Thermobifida halotorelans*, *Thermomyces lanuginosus*, *H. insolens* (Xyl43A), and *Enterobacter* sp. Conversely, β-xylosidases from *Paecilomyces thermophila* and *H. insolens* (Xyl43B) demonstrated better tolerance to xylose than did r*LfX*yl43. The tolerance of r*Lf*Xyl43 to NaCl was also determined (Figure 5B). Increasing concentration of NaCl did not affect the activity of r*Lf*Xyl43. The protein exhibited 100% activity at 100–1,000 mM NaCl, and 94% residual activity at 1,500 mM NaCl.

**TABLE 5:** Comparison of the xylose tolerance of recombinant β-xylosidase/α-L-arabinofuranosidase, r*Lf*Xyl43, from *Limosilactobacillus fermentum* SK152 with other characterized GH 43 enzymes with known *K*_i_ values.

Source	Enzyme	*K*_i_ (mM)	References
*Limosilactobacillus fermentum* SK152	*Lf*Xyl43	100.1	This study
*Lactobacillus brevis* ATCC 367	LbX	30.1	Jordan et al. (2013)
*Bacteroides ovatus*	BoXA	6.57	Jordan et al. (2017)
*Enterobacter* sp.	EcXyl43	79.9	Ontañon et al. (2018)
*Thermobifida halotolerans* YIM 90462T	ThXyl43	43.8	Yin et al. (2019)
*Humicola insolens* Y1	Xyl43 A/B	79/292	Yang et al. (2014)
*Thermomyces lanuginosus* CAU44	TlXyl43	63	Chen et al. (2012)
*Paecilomyces thermophila*	PtXyl43	139	Yan et al. (2008)
*Fusarium proliferatum*	-	5	Saha (2003b)

*K*_i_ is defined as the amount of xylose required to inhibit half of the β-xylosidase activity under the assay conditions.

### 3.7 Synergism of r*Lf*Xyl43 with xylanase

**FIGURE 6:**
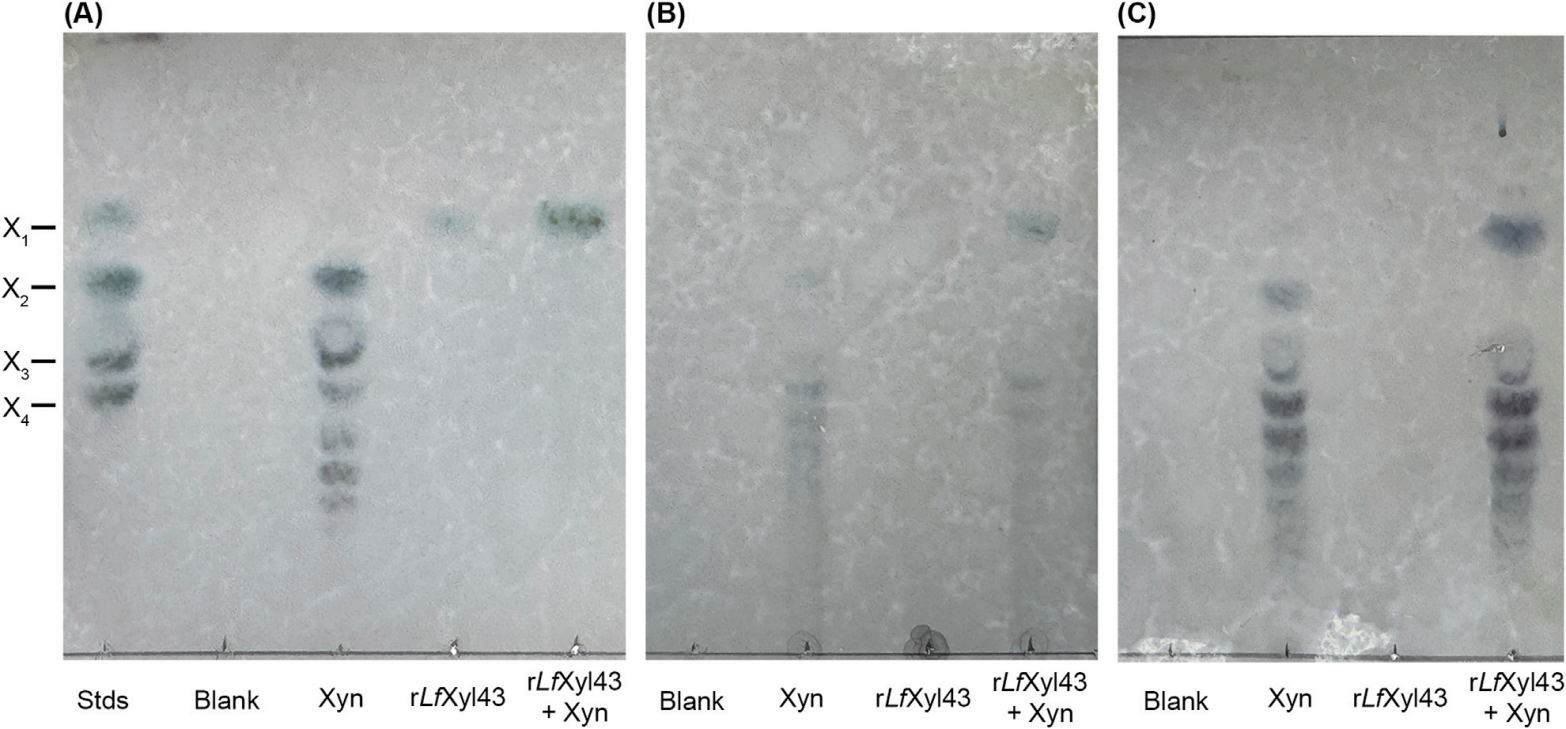
Synergism of the recombinant β-xylosidase/α-L-arabinofuranosidase, r*Lf*Xyl43, from *Limosilactobacillus fermentum* SK152 and a commercial xylanase. Thin layer chromatography analysis of the degradation of beechwood xylan **(A)**, rye arabinoxylan **(B)**, and wheat arabinoxylan **(C)** using 1 U/mL of r*Lf*Xyl43, commercial xylanase (Xyn), or both (simultaneous reaction). X_1_, xylose; X_2_, xylobiose; X_3_, xylotriose; and X_4_, xylotetraose.

The synergistic activity of r*Lf*Xyl43 with xylanases was investigated using a commercial endo-xylanase (Table 6). Equimolar amounts (1 U/mL each) of both r*Lf*Xyl43 and xylanase (Xyn) in simultaneous hydrolysis resulted in a modest degree of synergy towards beechwood xylan (1.33), rye arabinoxylan (1.2), and wheat arabinoxylan (1.2). TLC analysis (Figure 6) indicated the actions of individual enzymes against the substrates. For the three substrates, xylanase facilitated the endo-hydrolysis the xylan backbone producing shorter XOS, which were eventually hydrolysed by r*Lf*Xyl43 to produce xylose.

**TABLE 6:** Synergistic degradation of xylan and arabinoxylans by recombinant β-xylosidase/α-L-arabinofuranosidase, r*Lf*Xyl43, from *Limosilactobacillus fermentum* SK152, and a commercial xylanase (Xyn).

Enzymes	Beechwood xylan		Rye arabinoxylan		Wheat arabinoxylan	
	Xylose equivalents, mM	Degree of synergy	Xylose equivalents, mM	Degree of synergy	Xylose equivalents, mM	Degree of synergy
r*Lf*Xyl43	0.9 ± 0.1		0.03 ± 0.1		0.1 ± 0.1	
Xyn	7.12 ± 0.6		8.0 ± 0.2		8.2 ± 0.4	
r*Lf*Xyl43 + Xyn	10.8 ± 0.7	1.3	9.2 ± 0.7	1.2	9.4 ± 0.42	1.2

Values are reported as mean ± standard deviation of triplicate experiments. The degree of synergy was defined as the ratio of released xylose when the assay is performed with the two enzymes simultaneously to the sum of released xylose when the assay is performed with each enzyme alone.

## 4 Discussion

*Lm. fermentum* is an obligate hetero-fermentative LAB, typically associated with plant, dairy, and meat fermentation (Naghmouchi et al., 2020). Many of the *Lm. fermentum* strains are probiotic LAB, with anti-microbial and immuno-modulatory properties (Mikelsaar and Zilmer, 2009; Zhao et al., 2019; Yoo et al., 2023). Together with other LAB genera, *Lm. fermentum* has been reported to utilise XOS potentially due to the presence of multiple enzymes, including β-xylosidase (Ohara et al., 2006; Moura et al., 2007; Jang and Kim, 2011; Madhukumar and Muralikrishna, 2012; Michlmayr et al., 2012). However, as of date, β-xylosidase from *Lm. fermentum* has not been reported or characterised. Herein, we have characterised a putative β-xylosidase identified from the genome of *Lm. fermentum* SK152 isolated from kimchi. Preliminary analysis showed that *Lm. fermentum* SK152 can degrade xylan (Supplementary Figure S3). Genome annotation of *Lm. fermentum* SK152 revealed the presence of several GH enzymes, potentially used for the degradation of plant materials during fermentation or in the gut (Yoo et al., 2017). Analysis of the protein sequence of the putative β-xylosidase revealed that it belongs to the GH 43 subfamily 11. Furthermore, it showed sequence homology with other GH 43 subfamily 11 β-xylosidases, most notably with XynB1 of *Lv. brevis* ATCC 14869. β-Xylosidases in the GH 43 subfamily 11 are characterised by an N-terminal five-bladed β-propeller domain, where the catalytic domain can be found, and an auxiliary domain of β-sandwich domain at the C-terminus. Ontañon et al. (2018) demonstrated that, although the function of the auxiliary C-terminal domain is unclear, it is critical for the activity of the enzyme as some residues participate in its catalytic activity. Indeed, some residues found at the C-terminus of the *Lf*Xyl43 (Gly517, Glu518, Ile519, and Phe522) were predicted to be critical contact residues between *Lf*Xyl43 and its ligand. Similarly, amino acids Pro249, Asp250, Ala251, and Gly252 may also act as binding residues. Notably, these residues were only found between *Lf*Xyl43 and XynB1 of *Lv. brevis* ATCC 14869. These additional residues may offer advantages in terms of substrate specificity or stability but should be empirically determined in future investigations. Meanwhile, GH 43 enzymes have three highly conserved residues critical for their inverting function: an Asp, which acts as a general nucleophile/base, a Glu residue, which acts as a proton donor/acid, and another Asp residue, which acts as a p*K*a modulator of the proton donor. When compared with other characterised GH 43 β-xylosidase, *Lf*Xyl43 exhibited high structural homology with the β-xylosidase of *B. halodurans* C-125 (1YRZ), confirming that the putative enzyme belongs to the GH 43 family of enzymes. Furthermore, the catalytic residues of GH 43 enzymes are well conserved among GH 43 enzymes, including *Lf*Xyl43. Thus, we propose that the Asp15 and Glu188 residues of *Lf*Xyl43 act as general base and acid, respectively, whereas the Asp129 residue is a p*K*a modulator. The results of protein–ligand studies support this hypothesis, as ligands, such as pNPX, xylobiose, and xylotetraose, interacted with Asp15 and Glu188 residues at the −1 subsite.

The oligomeric state of β-xylosidase is also critical for its activity. Most characterised GH 43 β-xylosidases are homo-dimeric, homo-trimeric, or homo-tetrameric. The auxiliary C-terminal domain is believed to have a role in the formation of multimers in this enzyme family (Falck et al., 2016; Ontañon et al., 2018). Due to its high structural homology with other GH 43 β-xylosidases, we predicted that *Lf*Xyl43 might also have a multimeric state. Its closest homologue, 1YRZ, is characterised as homo-dimeric, whereas most of the GH 43 β-xylosidases, such as 8UWS, 3C2U, 2EXH, and 1YIF, are characterised as homo-tetrameric. Native PAGE analysis revealed that the recombinant *Lf*Xyl43 exists largely as a monomer, but can also exist as dimer, trimer, or tetramer. This is consistent with other data showing that β-xylosidase can exists as monomer or multimers in solution (Falck et al., 2016). The oligomeric state of β-xylosidases affects its functionality (Corradini et al., 2021); however, in the case of *Lf*Xyl43, it remains to be investigated.

For examining the biochemical characteristics and substrate specificities of r*Lf*Xyl43, the *Lfxyl43* gene was cloned and expressed in *E. coli*. The recombinant expression of *Lf*Xyl43 in the current study provided high enzyme yield and purity which is necessary for the characterization of the enzyme. The recombinant β-xylosidase showed high activity towards pNPX and oNPX, confirming its β-xylosidase activity. Activity towards oNPX among the GH 43 enzymes, especially those from LAB, has rarely been reported. r*Lf*Xyl43 also showed weak activity towards pNPAf, which indicates that it is a bi-functional enzyme. Other reported GH 43 enzymes have also demonstrated bi-functionality with higher activity as a β-xylosidase than as an α-L-arabinofuranosidase. For instance, XylBH43 from *B. halodurans* C-125, EcXyl43 from *Enterobacter* sp., BX43A from *Bifidobacterium animalis* BB-12, and XynB2 from *Lv. brevis* DSM 20054 exhibited higher catalytic efficiency towards pNPX than towards pNPAf (Liang et al., 2009; Michlmayr et al., 2013; Viborg et al., 2013; Ontañon et al., 2018). The bi-functionality of these enzymes may be attributable to the similarity in the spatial configuration of the −OH groups of D-xylose and L-arabinose (Liang et al., 2009). Regardless of the bi-functionality of r*Lf*Xyl43, its higher activity towards pNPX suggests that its major activity is as a β-xylosidase, rather than as a α-L-arabinofuranosidase. r*Lf*Xyl43 also exhibited high activity towards natural substrates, such as XOS and xylan. In fact, the steady-state kinetics of r*Lf*Xyl43 showed higher catalytic efficiency for XOS substrates than pNPX, with *k*_cat_/*K*_m_ values increasing in the order xylobiose < xylotriose < xylotetraose. Although belonging to the same GH family, not all GH 43 β-xylosidases exhibit similar catalytic efficiency towards XOS. For example, WXyn43 from *Weissella* sp. 92 exhibits higher catalytic efficiency towards xylobiose and xylotriose than towards xylotetraose (Falck et al., 2016). Conversely, increasing catalytic efficiency towards longer XOS was observed for BoXA (from *B. ovatus*), PcAxy43B (from *Paenibacillus curdanolyticus* B-6), EcXyl43A, and BXA43, similar to that for r*Lf*Xyl43 (Viborg et al., 2013; Jordan et al., 2017; Ontañon et al., 2018; Limsakul et al., 2021). Although it remains unclear as to why such phenomenon exists among enzymes of the same GH family, the presence of additional contact residues not present in other GH 43 enzymes may provide additional catalytic subsites that can accommodate longer substrates (Ontañon et al., 2018).

The effects of pH and temperature on the β-xylosidase/α-L-arabinofuranosidase activity of r*Lf*Xyl43 were also investigated in the present study. The activity of r*Lf*Xyl43 was highest at pH 7.0. This is consistent with reports for other GH 43 β-xylosidases from fungal and bacterial sources, for which the optimal pH ranges from 6 to 8. Recombinant β-xylosidase from *P. thermophila* (PtXyl43), *H. insolens* Y1 (Xyl43B), *T. halotolerans* YIM 90462^T^ (ThXyl43A), and *Bacillus pumilus* TCCC 11350 (Xyl) showed pH optima within this range (Yan et al., 2008; Yang et al., 2014; Liu et al., 2019; Yin et al., 2019). The optimal temperature for the activity of r*Lf*Xyl43 was found to be 35°C. r*Lf*Xyl43 also showed narrower pH and thermal stability compared with other GH 43 enzymes. The thermostability of r*Lf*Xyl43 was found to be much lower than that of other β-xylosidases (40°C–60°C) but is not uncommon among GH 43 β-xylosidases (Li et al., 2023). For example, β-xylosidases isolated from *B. ovatus* (BoXA) and *B. pumilus* TCCC 11350 (Xyl) exhibit optimal activity at 25°C and 30°C, respectively (Jordan et al., 2017; Liu et al., 2019). Cold-adapted enzymes are believed to form more flexible tertiary structures than those active at high temperatures (Liu et al., 2019; Li et al., 2023). The optimal activity of r*Lf*Xyl43 could potentially be related to niche adaptation to fermentation and gut environments, wherein the conditions are typically milder. This makes r*Lf*Xyl43 desirable for industrial degradation of lignocellulosic materials as it does not need high temperatures to retain its catalytic activity and would be suitable for a more sustainable and cost-effective process.

The effect of metal ions and other reagents on the activity of r*Lf*Xyl43 was also investigated. The activity of r*Lf*Xyl43 towards pNPX was slightly, but not dramatically, improved by the addition of Ca^2+^, Mn^2+^, and K^+^, and only at lower concentrations. At 10 mM, the addition of these ions, most notably by Ni^2+^, hindered the activity of r*Lf*Xyl43. Some GH 43 β-xylosidases are activated by the addition of divalent metal ions, such as Ca^2+^, Mg^2+^, and Mn^2+^. For instance, the β-xylosidases RS223-BX and RUM630-BX, isolated from cow rumen and wastewater metagenomic library, respectively, demonstrated enhanced catalytic activities in the presence of metal ions (Lee et al., 2013; Jordan et al., 2015). Moreover, a β-xylosidase from compost metagenome, CoXyl43, is also activated by Ca^2+^ ions (Matsuzawa et al., 2017). The β-xylosidase BoXA is also reported to be activated by Ca^2+^ (Jordan et al., 2017). These metal ion-activated β-xylosidases have metal-binding sites located at their catalytic N-termini. Whether *Lf*Xyl43 also has a metal-binding site similar to these β-xylosidases would need to be elucidated using crystallographic analysis.

During the hydrolysis of xylans by β-xylosidases, the concentration of monosaccharides, including xylose, increases which, in turn, inhibit the enzyme by blocking its catalytic pockets (Li et al., 2023). Despite this, some β-xylosidases showed exceptional tolerance to xylose. Bacterial β-xylosidases EcXyl43 from *Enterobacter* sp. and ThXyl43 from *T. halotolerans* are highly tolerant to xylose inhibition (*K*_i_ = 79.9 and 43.8, respectively) (Ontañon et al., 2018; Yin et al., 2019). β-xylosidases from fungal sources, such as Xyl43 A/B (from *H. insolens*), TlXyl43 (from *T. lanuginosus*), and PtXyl43 (from *P. thermophila*), also show high tolerance to xylose inhibition (*K*_i_ = 79/292, 63, and 139, respectively) (Yan et al., 2008; Chen et al., 2012; Yang et al., 2014). Notably, r*Lf*Xyl43 also demonstrated exceptional tolerance to xylose (*K*_i_ = 100.1). The *K*_i_ value of r*Lf*Xyl43 for xylose was found to be higher than that for previously reported β-xylosidases (except for Xyl43B and PtXyl43). Currently, there is limited data on xylose-tolerant β-xylosidases from LAB. The β-xylosidase from *Lv. brevis* ATCC 367, LbX, was found to have a *K*_i_ value of 30.1 for xylose (Jordan et al., 2013). In another study, Michlmayr et al. (2013) reported that XynB1 and XynB2 from *Lv. brevis* ATCC 14869 retained 80% and 33.8% of their respective activities at 100 mM xylose; however they did not report the *K*_i_ values for these enzymes. It has been shown that substrate affinity is directly linked to substrate inhibition. Previous studies have demonstrated that mutations in highly conserved residues Trp147 (in XylBH43 of *B. halodurans* C-125) and Trp145 (in SXA of *S. ruminantium*) to Gly147 and Gly145, respectively, resulted to lower substrate affinity and lower substrate inhibition (Fan et al., 2010; Wagschal et al., 2012). Although, the Trp147 residue of *Lf*Xyl43 is unaltered, neighbouring residues Glu146 and Glu148 (which are also present in XynB1, a potential xylose-tolerant β-xylosidase) could alter the interaction of the substrate at +1 subsite, decreasing its affinity. Moreover, to the best of our knowledge, no salt-tolerant β-xylosidase from LAB has been described as of date. The salt tolerance observed for r*Lf*Xyl43 may be due to the presence of highly negatively charged surface at the catalytic pocket (Supplementary Figure S4), which stabilizes binding of water molecules during protein-substrate interaction (Zhang et al., 2019; Wu et al., 2023; Cao et al., 2024). However, the tolerance mechanism of *Lf*Xyl43 towards salt and xylose must be validated through mutagenesis experiments. The tolerance of r*Lf*Xyl43 to high xylose and NaCl concentration offers promise for uses in industrial applications, mostly requiring enzymes from food-grade LAB.

Finally, in this study, we demonstrated that r*Lf*Xyl43 can hydrolyse xylans, specifically beechwood xylan, but not rye or wheat arabinoxylan. Although these xylans have similar 1,4-β-D-xylopyranoside backbones, rye and wheat arabinoxylan possess α-L-arabinofuranoside residues at either O-2 or O-3 positions unlike the 4-O-methylglucuronic acid substitution in beechwood xylan (Piro et al., 2023). Among the three xylans, rye is the most substituted, followed by wheat then beechwood–the differences in the degree of side-chain substitutions could influence the hydrolytic activity of xylanolytic enzymes (Rudjito et al., 2023). Despite exhibiting α-L-arabinofuranosidase activity, r*Lf*Xyl43 alone was not able to release xylose either from rye or wheat arabinoxylan indicating that r*Lf*Xyl43 cannot hydrolyse these linkages, which prohibits its access to the 1,4-β-D-xylopyranoside backbone. However, through synergistic activity with an endo-β-1,4-xylanase, r*Lf*Xyl43 contributed to the higher hydrolysis of these arabinoxylans to produce xylose (1.2-fold higher for both rye and wheat arabinoxylan). Several GH 43 β-xylosidases demonstrate synergy with xylanase. β-Xylosidase from *B. pumilus* TCCC 11350 exhibited a high degree of synergy with a xylanase during simultaneous degradation of beechwood xylan (Liu et al., 2019). Additionally, EcXyl43 also demonstrated cooperative activity with a xylanase in degrading xylan (Ontañon et al., 2018), comparable to the activity of r*Lf*Xyl43. This further underscores the potential applicability of r*Lf*Xyl43 in the degradation of hemicellulose for industrial purposes.

*Lf*Xyl43 is a β-xylosidase belonging to the GH 43 subfamily 11, isolated from *Lm. fermentum* SK152. It showed high homology with other GH 43 β-xylosidases. We found that r*Lf*Xyl43 exhibited high activity towards pNPX, XOS, and xylan, and weak α-L-arabinofuranosidase activity towards pNPAf. The optimal pH and temperature for the activity of r*Lf*Xyl43 were determined to be 7.0°C and 35°C, respectively. r*Lf*Xyl43 showed synergism with an endo-xylanase in degrading xylans and arabinoxylans. Finally, r*Lf*Xyl43 was found to be tolerant to high concentrations of NaCl and xylose, similar to other xylose-tolerant β-xylosidase reported previously. To our knowledge, *Lf*Xyl43 is the first salt and xylose-tolerant β-xylosidase characterised from this LAB genus. Overall, this new β-xylosidase is a potential candidate enzyme for the degradation of xylan hemicelluloses that could be exploited for industrial and biomedical applications.

## Data Availability

The datasets presented in this study can be found in online repositories. The names of the repository/repositories and accession number(s) can be found in the article/Supplementary Material.
