# Supplementary material for: Heterologous expression and characterization of xylose-tolerant GH 43 family β-xylosidase/α-L-arabinofuranosidase from Limosilactobacillus fermentum and its application in xylan degradation
Source: Front Bioeng Biotechnol. 2025 Mar 10;13:1564764. doi: 10.3389/fbioe.2025.1564764 (PMC11931166; doi:10.3389/fbioe.2025.1564764)
Supplement: Supplementary file 1 [file DataSheet1.pdf]

SUPPLEMENTARY INFORMATION

Heterologous expression and characterization of xylose-tolerant GH 43 family β-xylosidase/α-L-arabinofuranosidase from *Limosilactobacillus fermentum* and its application in xylan degradation

Robie Vasquez[1], Ji Hoon Song[1], Jae Seung Lee[1], Sanghoon Kim[1†], and Dae-Kyung Kang[1]*

[1] Department of Animal Biotechnology, Dankook University, Cheonan, 31116, Republic of Korea

Present address:
[†] College of Pharmacy and Pharmaceutical Sciences, Florida A&M University, Tallahassee, FL, 32301, USA

Supplementary Table S1. List of buffers used to determine the effect of pH on the activity of β-xylosidase/α-L-arabinofuranosidase r*Lf*Xyl43 from *Limosilactobacillus fermentum* SK152.

Buffer	pH
50 mM potassium chloride buffer	2.0–3.0
50 mM sodium acetate buffer	4.0–5.0
50 mM sodium phosphate buffer	6.0–8.0
50 mM Tris-Cl buffer	9.0–10.0

```
ATGAAAACTA TCCAAAATCC GATTATTCCG GGGATGGCTC CCGATCCATC AATTATTCGG 60
GTAGGGGACG ATTATTACAT TGCTACTTCC ACCTTTCACT GGAAGCCAGC AGTCCAGATC 120
TTTCACTCAC GGGATTTAGC CAACTGGGAA TTAGTTTCGT ATGGGTTGAG TAAAGATGAA 180
GTTGACTTAC GGGGTACCAA CACGCCGGCG GGAATTTGGG CGCCCCACCT GTCTTATGAC 240
GAAAAGACTA AGCGTTTTTG GCTGGTTTAT TCCCACATGC AAAACATGGC GGGACGAGAA 300
TTTAACGCAG AATCGTATGC GATGTCGGCG GATGAGATTA CTGGACCATG GTCAAAGCCC 360
GTTTACCTAA CCTCAATTGG TTTTGACCCG GCACTTTTCC ACGATCAAGA CGGGCGCCAT 420
TACCTATCGA TTTTGGAATG GGAAACTCGT CAGGGCTACC AAGCGCCAGG ACACATTGTG 480
ATCGCTGAAG TTGATTTGAC AACGGGCCAA GTTGGCGAGT GGCATCGGGT CACAACCGGG 540
TTTACCACCC GCGGGTGCGT GGAAGCCCCG CAGCTTTACC GTCATGGTGA TTACTACTAC 600
TTGTTGCTTG CCTCAGGGGG AACCGGGTAT GGCCACGGAA TTGAAATTGG CCGTAGTAAA 660
CAGGTCTTTG GCCCTTACGA ACCGGATCCA AGTGGGGAAC CAATTCTAAC GTCACAACCA 720
GCGCACTTAT TCTCGTTGGG CGATCCGGAT GCGGGGCACT TTGAGATGTA CAATCCGCAC 780
TCGGAAATGC AAAAGGCGGG CCACGGTTCG CTAGTTGAAA CGAAAGACGG TGAGTGGTAC 840
CTAGCCCACC TGATGGCGCG GCCACTCCAG GGGAAGTTAC TTAACCCATT GGGGAGGGAG 900
ACGTCAATTC AAAGAGTGGA CTGGAATGAT GAGGGATGGC TTAGGCTACA TGATGGGTCG 960
AATGTAGCGA AGATGACCAC CCCCGCCCCG GCGGCCTTTA ATGGTTCACA AAAGTCAGCT 1020
GCCTTTGACA TTGTGGATGA TTTTACTTCA CCGACTTTAA ATATCCGTTA CATGACCCCG 1080
TACCAAGAAC CAGCTAGTTC GTGGATTGAC TACCAGGGGA ACGGAAAGTT AAAAATTAAG 1140
GGGCGCAATT CATTTTTCTC CCAGTACGCA CCGTCAATTC TTGCCACTAG GGCTACCTCA 1200
TTGAACTACG AAGTCGAAAC GGAACTGGAA TTTCACCCCG ATCATTATTC AGAAACGGCC 1260
GGTCTCGGCC TATACTATGA CGCAAATAAT TGGTTCTACG TTCGCTTATG CCTAGCTAAT 1320
GATGAACAAG GGATTGTCTT GTGTGGCTTG CAGGCGAAAC TGGGGCAAAA AATCGATGAT 1380
CGTGATAATG AAGTTGTCGT CCCCGGCGGG AAGGTAAAAG TTCGTTTGAG TTACCGACAG 1440
GGGATGGTTG AGGTTGATTA CCAACTTCCT GAGCAAGAAG GATGGCAGCC ACTAGGAGGA 1500
CCAGTCAACG TTGATTATCT TTCGGATGAA GGGGTTAACG GGGAACCTGG CGAAATTGGT 1560
GGTTTCACCG GTCTATTTAA TTTTATGGGG GCCGTTGATG CCCACCAGCA TGATTCCGTT 1620
GCAACGTTTA ACTACTACCG GGTAACGAGC CGGTAA 1656
```

Supplementary Figure S1. Nucleotide sequence of the β-xylosidase/α-L-arabinofuranosidase gene, *Lfxyl43,* from *Limosilactobacillus fermentum* SK152.

```
  1 MKTNG MADSR VGDDY YATST HWKAV HSRDA
 31 NWVSY GSKDV DRGTN TAGWA HSYDK TKRWV
 61 YSHMN MAGRN ASYAM SADTG WSKVY TSGDA
 91 HDDGR HYSWT RGYAG HVAVD TTGVG WHRVT
121 TGTTR GCVAY RHGDY YYASG GTGYG HGGRS
151 KVGYD SGTSA HSGDD AGHMY NHSMK AGHGS
181 VTKDG WYAHM ARGKN GRTSR VDWND GWRHD
211 GSNVA KMTTA AANGS KSAAD VDDTS TNRYM
241 TYASS WDYGN GKKKG RNSSY ASATR ATSNY
271 VTHDH YSTAG GYYDA NNWYV RCAND GVCGA
301 KGKDD RDNVV VGGKV KVRSY RGMVV DYGWG
331 GVNVD YSDGV NGGGG TGNMG AVDAH HDSVA
361 TNYYR VTSR
```

Supplementary Figure S2. Amino acid sequence of the β-xylosidase/α-L-arabinofuranosidase *Lf*Xyl43, from *Limosilactobacillus fermentum* SK152. GenBank Accession No. PQ818275.

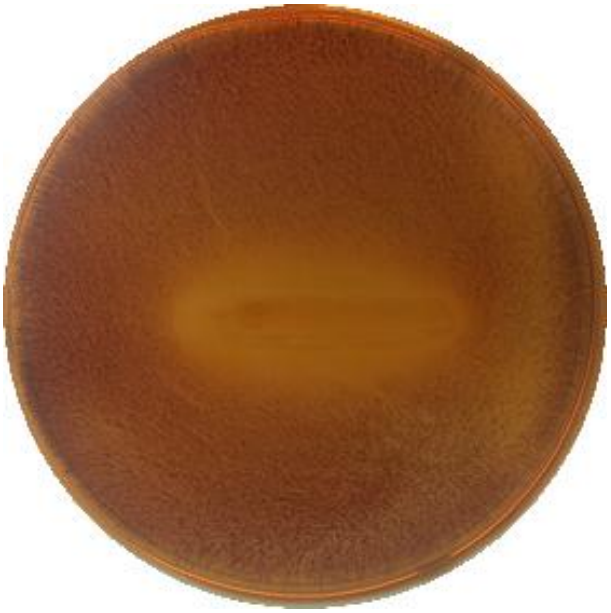

Supplementary Figure S3. Growth of *Limosilactobacillus fermentum* SK152 on Man Rogosa Sharpe agar supplemented with 1% (w/v) xylan substrate. Zone of clearance indicates activity towards the substrate.

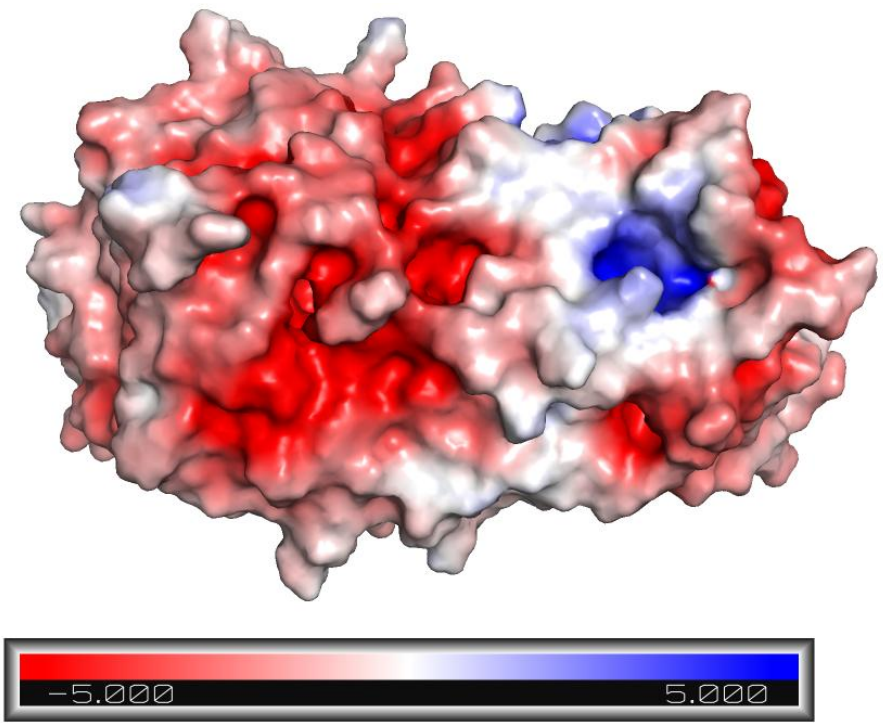

Supplementary Figure S4. Electrostatic potential of the structural surface of *Lf*Xyl43. Positive charges are shown in blue, and negative charges are shown in red.
